# Comparison of the safety and efficacy of EDUG-guided tunnelled PICC versus traditional PICC in patients with cancer: A retrospective study

**DOI:** 10.1097/MD.0000000000044727

**Published:** 2025-10-10

**Authors:** Juan Zeng, Yi Zhang, Huili Xu

**Affiliations:** a Oncology Department, Wuhan Central Hospital, Wuhan, Hubei Province, China.

**Keywords:** cancer patients, catheter-related infection, EDUG guidance, indwelling time, PICC, retrospective study, tunneled pathway, venous thrombosis

## Abstract

Peripherally inserted central catheters (PICC) are widely used for intravenous therapy in patients with cancer. However, traditional PICC placement is associated with a lower first-attempt success rate, a higher risk of catheter-related infections, and challenges in catheter maintenance. In recent years, the echo-dynamic ultrasound-guided (EDUG) tunneled PICC, a modified technique, has been increasingly adopted in clinical practice. This study aims to compare the safety and efficacy of EDUG-guided tunneled PICC with traditional PICC in cancer patients. A retrospective analysis was conducted using medical records of 156 cancer patients who underwent PICC placement at our hospital between January 2022 and December 2024. Patients were categorized into the EDUG group (n = 79) and the traditional PICC group (n = 77). Data were collected from electronic health records, and key outcomes compared between the 2 groups included first-attempt success rate, catheter-related infections, catheter-associated venous thrombosis, local complications, and indwelling time. Baseline characteristics were comparable between the 2 groups (all *P* > .05). The first-attempt success rate was significantly higher in the EDUG group (96.2%) compared to the traditional group (85.7%) (*P* = .033). The incidence of catheter-related infections was lower in the EDUG group (2.53% vs 7.79%, *P* = .045). Although the rate of venous thrombosis was not statistically different (1.27% vs 6.49%, *P* = .060), no significant difference was observed between the 2 groups. The overall complication rate was significantly reduced in the EDUG group (5.1% vs 14.3%, *P* = .029). Moreover, the average indwelling time was significantly longer in the EDUG group (96.8 ± 22.3 days vs 83.6 ± 19.5 days, *P* < .001). EDUG-guided tunneled PICC demonstrates a higher success rate, lower infection and complication rates, and a longer indwelling time compared with traditional PICC in cancer patients. These findings suggest that EDUG-guided tunneled PICC may offer clinical advantages over traditional PICC, particularly in terms of catheter stability and reduced complications. Further studies are needed to confirm these benefits and assess their generalizability. This technique offers favorable safety and stability and is suitable for clinical application in patients requiring long-term intravenous therapy.

## 1. Introduction

Catheter-related complications remain a significant challenge in intravenous therapy for cancer patients. Peripherally inserted central catheters (PICC), a commonly used central venous access device, are favored for their ease of insertion, extended dwell time, and broad clinical indications, particularly for patients undergoing chemotherapy, parenteral nutrition, or targeted therapies.^[1–4]^ Despite their widespread use, traditional PICC placement still presents several clinical challenges, including operator-dependent variability in first-attempt success, increased risk of catheter dislodgement or exposure, and a higher incidence of catheter-related bloodstream infections (CRBSI) and venous thrombosis.^[5–7]^ These complications not only affect patient quality of life and treatment adherence but also place additional strain on healthcare professionals. Furthermore, cancer patients are more vulnerable to infections and complications due to factors like chronic malnutrition and immunosuppression, which demand higher standards for catheter safety and stability.^[8,9]^

In response to these challenges, the Echo-Dynamic Ultrasound-Guided (EDUG) tunneled PICC has emerged as a promising alternative. This technique combines ultrasound-guided vascular access with subcutaneous tunneling to enhance catheter fixation, reduce exit-site tension, and decrease the likelihood of bacterial colonization.^[10–12]^ It has been shown that tunneled PICCs offer lower infection and dislodgement rates, particularly in intensive care settings or in patients with hematological diseases.^[13–15]^ Despite these advantages, data comparing EDUG tunneled PICCs with traditional PICCs in cancer patients, especially concerning intraoperative success, thrombotic risks, and long-term catheter performance, remain limited.

The present study builds on previous work by focusing on a cancer patient population from a single institution, providing insight into the long-term outcomes associated with EDUG-guided tunneled PICCs. Unlike many prior studies, which primarily report short-term outcomes or focus on specific cancer types, our research provides detailed classification of complications such as catheter-related infections, venous thrombosis, local complications, and indwelling time.^[16,17]^ Additionally, our study includes a follow-up period extending over several months (2022–2024), allowing us to assess long-term catheter performance. This study is distinctive in its approach by directly comparing the EDUG-guided tunneled PICC technique with traditional PICC placement in an oncology cohort, emphasizing practical clinical outcomes that can guide future clinical practice.

To address this gap, we conducted a retrospective study involving cancer patients who underwent PICC placement at our institution between January 2022 and December 2024. Our aim was to compare EDUG-guided tunneled PICCs with traditional PICCs across key clinical outcomes, including first-attempt success rate, catheter-related infections, catheter-associated venous thrombosis, local complications, and indwelling time. The findings from this study will provide evidence-based insights to optimize central venous access strategies for oncology patients, and contribute to the broader implementation of EDUG technology in intravenous oncology care.

## 2. Methods

### 2.1. Study design and participants

This study was approved by the Ethics Committee of Wuhan Central Hospital. This retrospective controlled study included 156 hospitalized cancer patients who underwent PICC placement at our institution between January 2022 and December 2024. Based on the type of catheter placement, patients were divided into 2 groups: the EDUG-guided tunneled PICC group (EDUG group, n = 79) and the traditional PICC group (n = 77). All patients were approved by the institutional ethics committee, and their clinical data were complete and traceable. Inclusion criteria were: confirmed diagnosis of malignancy; requirement for long-term intravenous infusion therapy; first-time PICC placement. Exclusion criteria included: history of central venous stenosis or catheter implantation; intolerance to the procedure due to coagulation dysfunction; incomplete follow-up data or catheter indwelling time <7 days.

### 2.2. PICC placement procedure

In the EDUG group, ultrasound guidance combined with guidewire assistance was used to puncture upper arm veins, followed by the creation of a subcutaneous tunnel to relocate the catheter exit site to the distal upper arm or medial forearm to reduce skin tension and infection risk. In the traditional PICC group, standard puncture and external catheter fixation methods were applied. All procedures were performed under aseptic conditions by a team of 4 experienced operators, each with at least 5 years of experience in PICC placement.

To ensure operational consistency, all operators followed established standard operating procedures for catheter insertion and care. Additionally, regular training and calibration sessions were conducted to maintain skill levels and minimize inter-operator variability. Catheter tip position was confirmed by postoperative chest X-ray.

### 2.3. Data collection and observation indicators

Baseline data collected for both groups included demographic characteristics (age, sex, height, weight, BMI, blood pressure, heart rate), clinical information (tumor type, receipt of chemo/radiotherapy), laboratory parameters (PT, APTT, platelet count, fasting blood glucose), and smoking history. The primary outcome measures were: first-attempt success rate of catheter placement; incidence of catheter-related bloodstream infection (CRBSI); incidence of catheter-associated venous thrombosis; incidence of catheter-related discomfort and local complications (e.g., dislodgement, insertion site oozing, skin reactions); indwelling time (in days).

### 2.4. Definitions and diagnostic criteria for complications

The diagnosis of CRBSI was based on CDC criteria, requiring both positive blood cultures and clinical evidence of infection, with exclusion of other infection sources. Catheter-related thrombosis was confirmed by Doppler ultrasonography. Local complications included catheter dislodgement, insertion site exudation, erythema, pruritus, or allergic reactions, all confirmed through nursing records and retrospective chart review.

### 2.5. Sample size calculation

A sample size calculation was performed prior to the study to ensure sufficient power to detect significant differences in the primary outcomes. Based on previous studies that compared EDUG-guided tunneled PICC with traditional PICC in similar populations, it was estimated that a minimum of 70 patients per group would provide adequate power (80%) to detect a significant difference in key clinical outcomes, such as first-attempt success rate and catheter-related infections, with an alpha level of 0.05. The sample size of 156 patients (79 in the EDUG group and 77 in the traditional group) was based on the available data from our institution, and was found to be sufficient to observe clinically meaningful differences in the outcomes assessed in this study.

### 2.6. Statistical analysis

Data were analyzed using SPSS version 26.0 (Chicago). Continuous variables were expressed as mean ± standard deviation and compared using the independent-samples t-test. Categorical variables were expressed as frequencies and percentages, and compared using the chi-square (χ²) test. A *P*-value <.05 was considered statistically significant.

## 3. Result

### 3.1. Comparison of baseline characteristics

A total of 156 cancer patients who underwent PICC placement at our hospital between January 2022 and December 2024 were included in this study. Based on the catheterization method, patients were divided into 2 groups: the EDUG-guided tunneled PICC group (EDUG group, n = 79) and the traditional PICC group (n = 77). Demographic and clinical characteristics were statistically compared between the 2 groups. We performed statistical adjustments for potential confounding factors such as age, gender, BMI, heart rate, systolic and diastolic blood pressure, smoking history, fasting blood glucose, tumor type, and coagulation parameters (PT, APTT, platelet count). The results showed no statistically significant differences in age, sex, primary tumor type, body mass index (BMI), receipt of chemo/radiotherapy, coagulation parameters (PT, APTT), platelet count, height, weight, heart rate, systolic and diastolic blood pressure, smoking history, or fasting blood glucose (all *P* > .05), indicating good baseline comparability between groups. Detailed data are presented in Table 1.

**Table 1:** Comparison of baseline characteristics between the 2 groups.

Variable	EDUG group (n = 79)	Traditional PICC group (n = 77)	t/χ² value	*P*-value
Age (year)	59.4 ± 11.2	58.9 ± 10.6	0.276	.783
Gender (male/female)	41/38	40/37	0.004	.95
Height (cm)	165.8 ± 7.9	164.9 ± 8.1	0.69	.492
Weight (kg)	62.4 ± 10.3	63.0 ± 9.8	−0.364	.716
BMI (kg/m²)	22.6 ± 3.4	23.0 ± 3.6	−0.728	.468
Heart rate (beats/min)	76.3 ± 9.2	77.1 ± 8.8	−0.538	.592
Systolic blood pressure (mm Hg)	126.5 ± 15.4	128.0 ± 14.7	−0.621	.535
Diastolic blood pressure (mm Hg)	78.4 ± 10.2	79.7 ± 9.5	−0.811	.419
Smoking history (yes/no)	18/ 61	20/ 57	0.203	.652
Fasting blood glucose (mmol/L)	5.7 ± 1.3	5.8 ± 1.4	−0.392	.695
Tumor type (solid/hematologic)	67/ 12	65/ 12	0.002	.961
Chemoradiotherapy (yes/no)	58/ 21	55/ 22	0.071	.79
Prothrombin time, PT (s)	11.4 ± 1.1	11.5 ± 1.0	−0.504	.615
Activated partial thromboplastin time, APTT (s)	30.2 ± 3.8	29.8 ± 3.5	0.633	.528
Platelet count (×10⁹/L)	212.5 ± 54.6	219.3 ± 49.8	−0.827	.41

### 3.2. Comparison of first-attempt success rate

**Figure 1.**
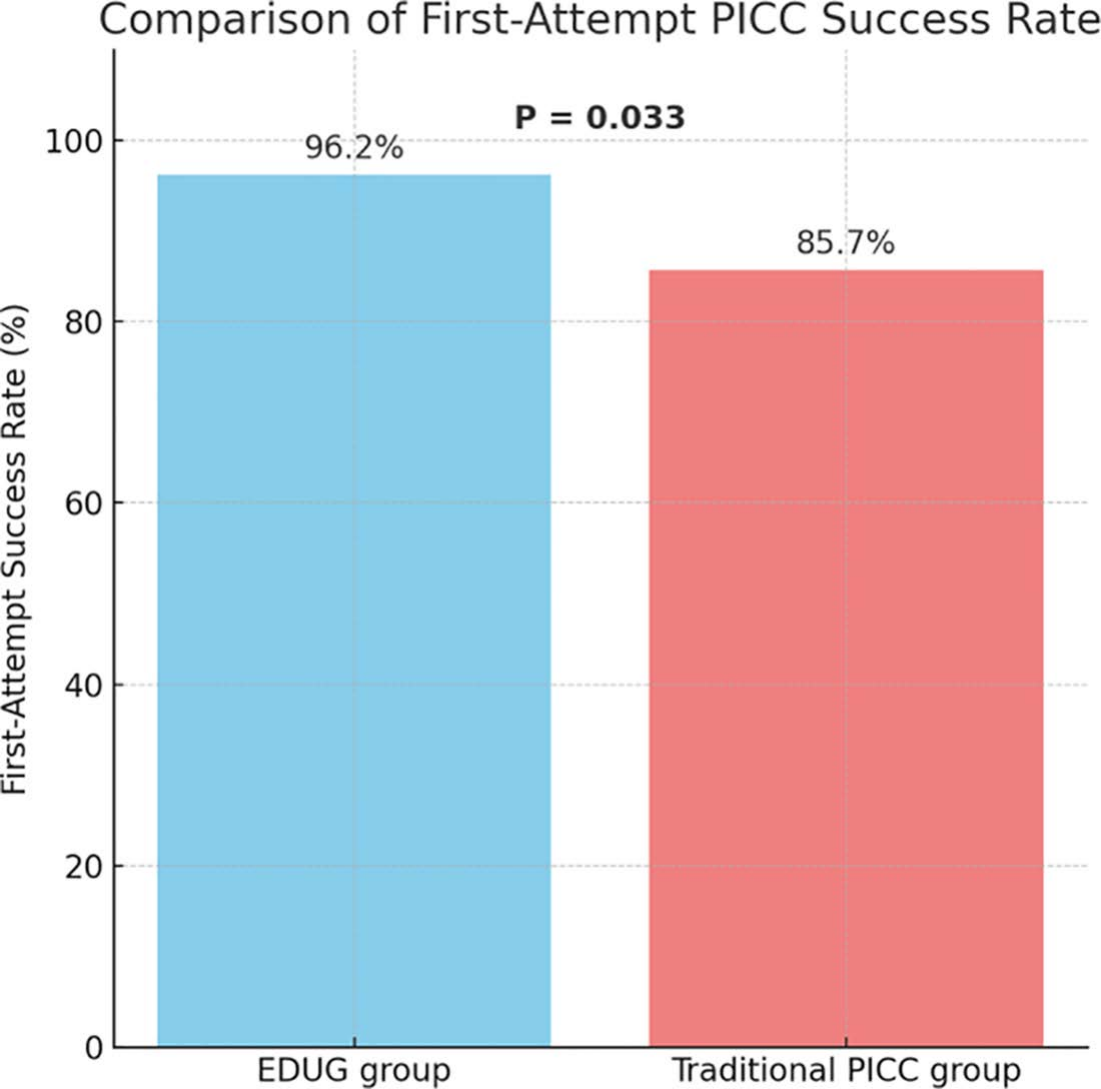
Comparison of first-attempt PICC success rate. PICC = peripherally inserted central catheters.

In this study, the first-attempt success rate of catheter placement was significantly higher in the EDUG-guided tunneled PICC group compared to the traditional PICC group. In the EDUG group, 76 out of 79 patients (96.2%) achieved successful catheterization on the first attempt, whereas the success rate in the traditional group was 85.7% (66/77). The difference between the 2 groups was statistically significant (χ² = 4.52, *P* = .033), as shown in Figure 1. These findings suggest that EDUG guidance can significantly improve the success rate of initial puncture, reduce the need for repeated attempts, enhance patient comfort, and potentially lower the risk of procedure-related complications.

### 3.3. Comparison of catheter-related bloodstream infection rate

**Figure 2.**
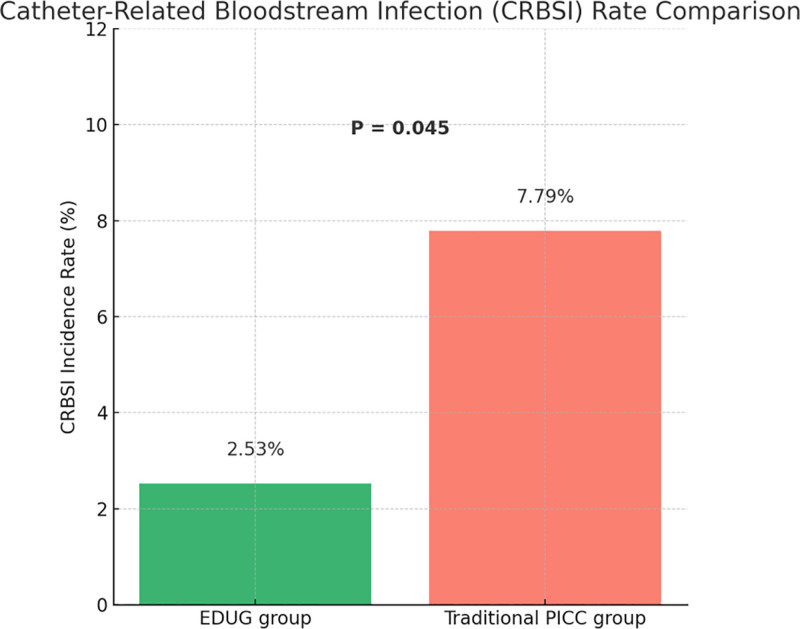
CRBSI rate comparison. CRBSI = catheter-related bloodstream infection.

During the follow-up period, catheter-related bloodstream infections (CRBSIs) occurred in 2 patients (2.53%) in the EDUG group, compared to 6 patients (7.79%) in the traditional PICC group. The difference between the 2 groups was statistically significant (χ² = 4.01, *P* = .045), as shown in Figure 2. These findings suggest that the EDUG-guided tunneled PICC approach can significantly reduce the risk of catheter-related infections. This advantage may be attributed to the increased stability of the subcutaneous tunnel, which reduces catheter dislodgement and local skin tension, thereby enhancing the long-term safety of PICC use.

### 3.4. Comparison of catheter-associated venous thrombosis rate

**Figure 3.**
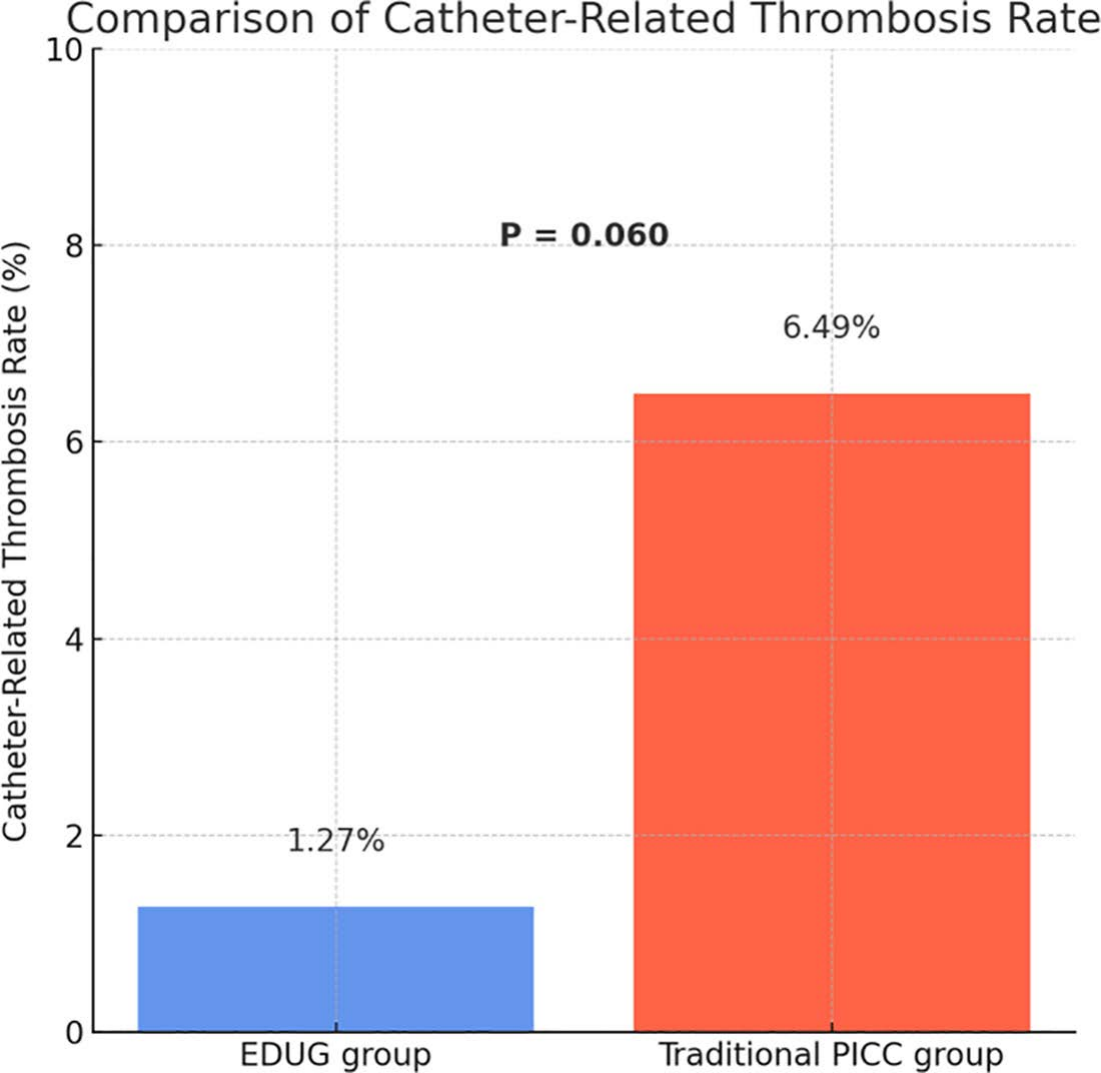
Comparison of catheter-related thrombosis rate.

During the study period, catheter-associated venous thrombosis occurred in only 1 patient (1.27%) in the EDUG group, compared to 5 patients (6.49%) in the traditional PICC group. The difference between the 2 groups approached statistical significance (χ² = 3.54, *P* = .060), as shown in Figure 3. Although the result did not reach conventional levels of statistical significance, a clearly lower incidence of thrombosis was observed in the EDUG group, indicating a downward trend. This finding suggests that the EDUG-guided tunneled PICC technique may offer advantages in optimizing catheter positioning and reducing irritation to the vascular endothelium, thereby potentially lowering the risk of thrombosis – an aspect worthy of further investigation in future studies.

### 3.5. Comparison of catheter-related discomfort and local complication rates

**Figure 4.**
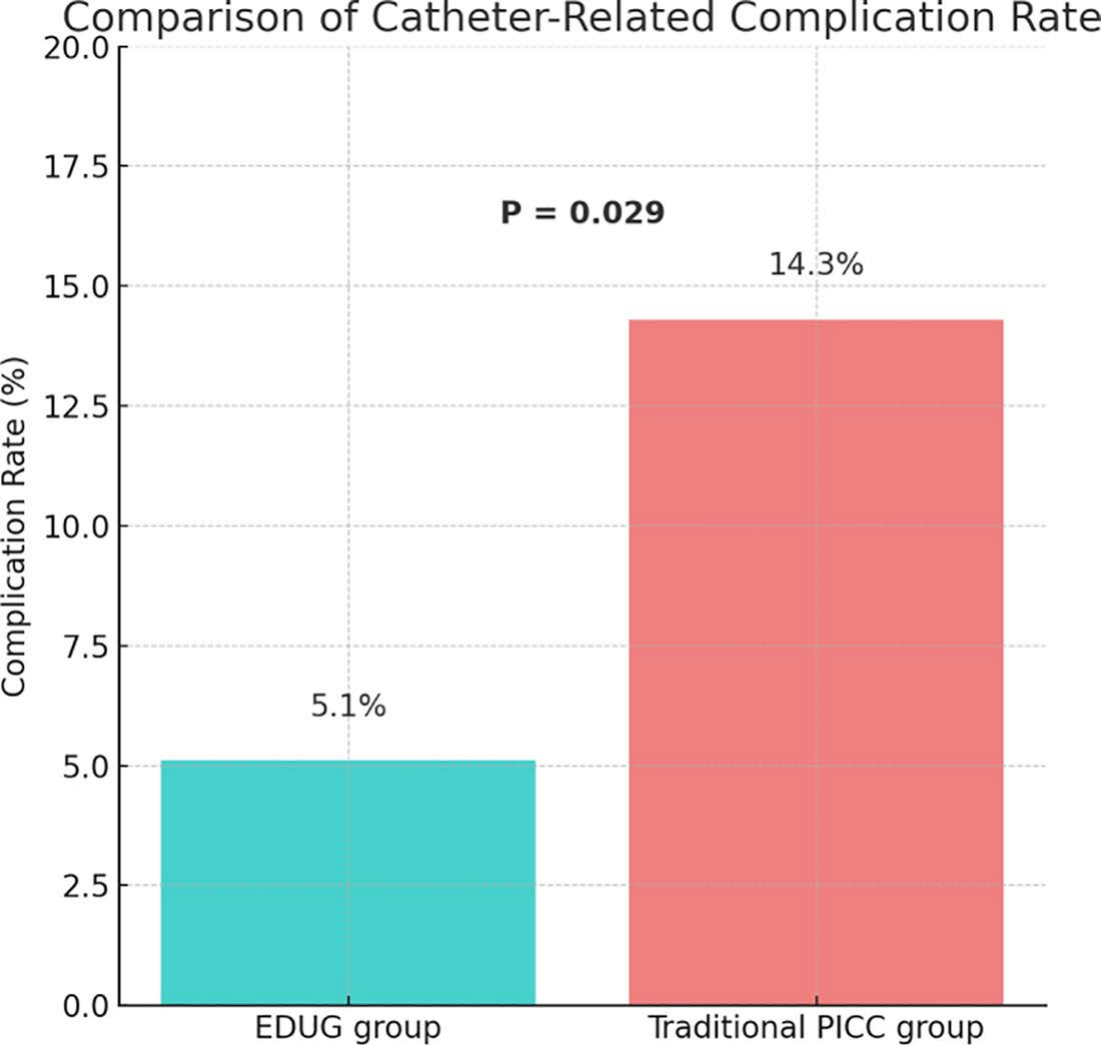
Comparison of catheter-related complication rate.

As shown in Figure 4, a total of 4 patients (5.1%) in the EDUG group experienced catheter-related discomfort or local complications, including catheter dislodgement, insertion site exudation, and local skin hypersensitivity. In contrast, 11 patients (14.3%) in the traditional PICC group developed such complications. The difference between the 2 groups was statistically significant (χ² = 4.79, *P* = .029). These findings suggest that the EDUG tunneled pathway not only enhances subcutaneous catheter stability and reduces discomfort due to catheter traction, but also helps to minimize skin tension and exudation at the insertion site. This may confer superior biocompatibility and patient tolerance during long-term catheter use.

### 3.6. Comparison of indwelling time

**Figure 5.**
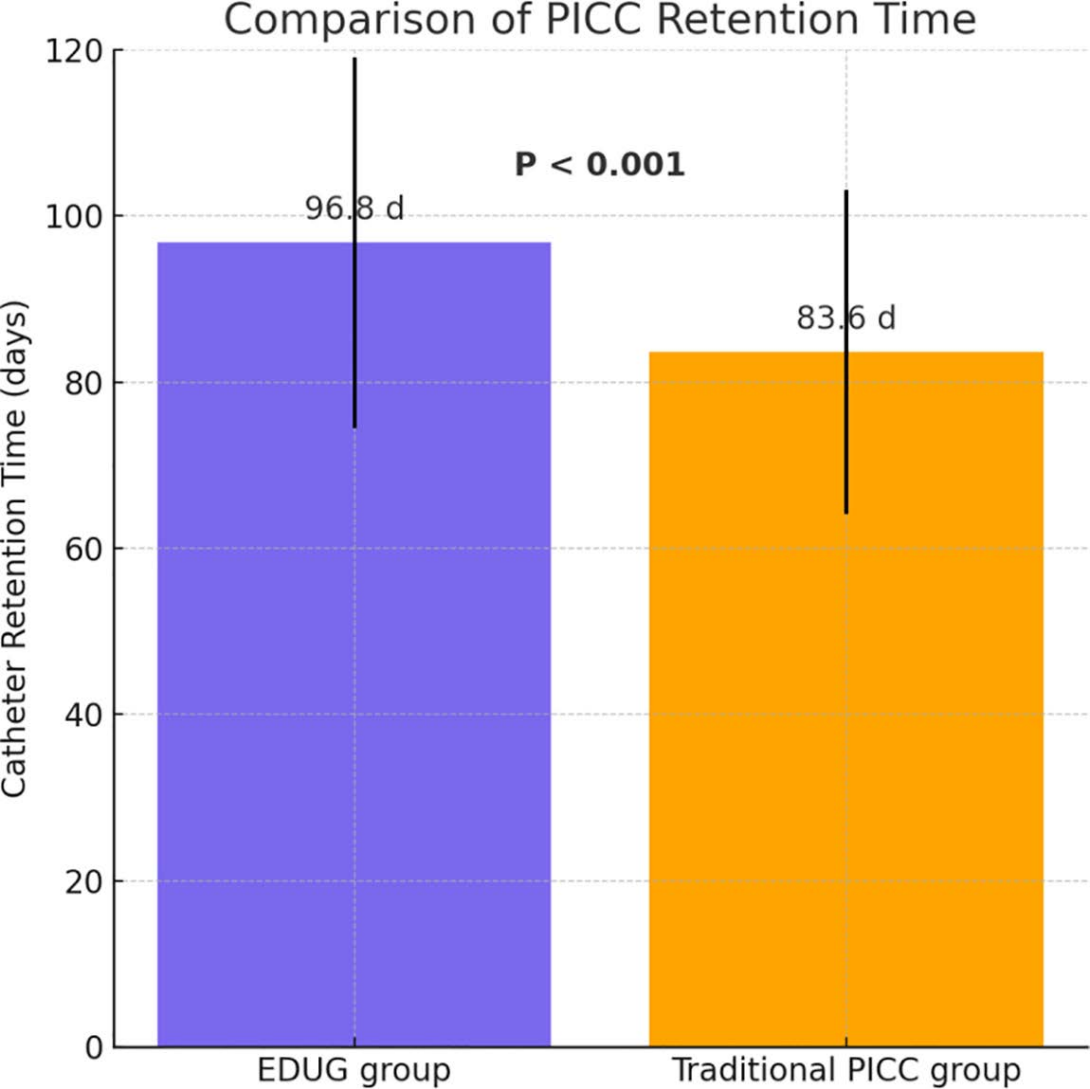
Comparison of PICC retention time. PICC = peripherally inserted central catheters.

Indwelling time was compared between the 2 groups. The results showed that the average indwelling time in the EDUG group was significantly longer (96.8 ± 22.3 days) than that in the traditional PICC group (83.6 ± 19.5 days). The difference was statistically significant according to the independent-samples *t*-test (*t* = 4.12, *P* < .001), as illustrated in Figure 5. This finding indicates that the tunneled pathway established under EDUG guidance offers superior catheter stability, effectively reduces catheter-related complications, and prolongs indwelling time. Therefore, it is particularly suitable for cancer patients requiring long-term intravenous therapy.

## 4. Discussion

PICCs, a form of central venous access, are widely used in cancer patients for chemotherapy, nutritional support, and intravenous therapy. However, traditional PICC placement methods are associated with several clinical challenges, including catheter dislodgement, a relatively high infection rate, low first-attempt success rate, and limited indwelling time.^[18]^ In recent years, the EDUG tunneled PICC has emerged as a novel technique that potentially improves catheter stability, extends catheter lifespan, and reduces complications.^[19]^

This retrospective study included 156 cancer patients who underwent PICC placement at our institution between 2022 and 2024. Patients were divided into 2 groups based on the catheterization method – EDUG-guided tunneled PICC (n = 79) and traditional PICC (n = 77). The baseline characteristics between the 2 groups were statistically comparable, ensuring the methodological validity of the subsequent analysis. The results demonstrated that the EDUG group outperformed the traditional group in several key metrics, including first-attempt success rate, incidence of catheter-related infections and complications, and indwelling time. These findings suggest that the EDUG-guided tunneled pathway offers substantial clinical advantages in oncology populations.

Previous studies have also supported the utility of EDUG and similar modified PICC techniques in enhancing placement success and reducing infection rates.^[20]^ For example, ultrasound-guided tunneled PICCs have been reported to achieve a first-attempt success rate of over 95%, significantly higher than that of traditional methods.^[21]^ Additionally, the tunneled approach has been shown to reduce tension at the catheter exit site, decrease local exudation, and minimize skin irritation, thereby reducing the risk of local complications.^[22]^ Building on these findings, our study further confirms the benefits of tunneled PICCs in reducing thrombotic risk and enhancing long-term catheter stability, especially in cancer patients who rely heavily on durable venous access for ongoing treatment.

In our cohort, the EDUG group achieved a first-attempt success rate of 96.2%, significantly higher than the 85.7% observed in the traditional group. This may be attributed to better alignment with vascular anatomy and more accurate guidewire manipulation afforded by the tunneled technique. The CRBSI rate was 2.53% in the EDUG group, markedly lower than the 7.79% in the traditional group, possibly due to the extended subcutaneous catheter path, which increases the distance between the skin exit site and the vessel, reducing the likelihood of bacterial colonization. Although the difference in catheter-associated venous thrombosis did not reach statistical significance, the EDUG group showed a clear downward trend, suggesting potential benefits in maintaining endothelial integrity and hemodynamic flow.

Importantly, the mean indwelling time in the EDUG group was 96.8 days, significantly longer than the 83.6 days in the traditional group. This reflects better catheter retention and patient compliance. For cancer patients undergoing prolonged chemotherapy and complex intravenous treatment regimens, longer catheter lifespan is critical for ensuring continuity of care and maintaining quality of life.

Compared to previous studies, our work offers several novel contributions: First, it is the first to systematically compare EDUG tunneled PICC versus traditional PICC across multiple clinical endpoints in a Chinese oncology population, including detailed classification of complications such as catheter dislodgement, insertion site leakage, and localized skin reactions, enhancing the interpretability of our findings. Second, the use of real-world, retrospectively collected data with a moderate sample size and well-matched groups adds robustness to the conclusions. Third, we used graphical statistical representations to clearly illustrate intergroup differences and P-values, improving the clarity and clinical applicability of the results.

While the EDUG-guided tunneled PICC technique demonstrates promising clinical outcomes, it is important to consider the cost-benefit and operational complexity of this approach for broader clinical application. The use of ultrasound guidance and subcutaneous tunneling may require more specialized equipment and training, which could increase the initial cost compared to traditional PICC placement methods. Additionally, the time and resources needed for training operators and maintaining consistency in procedures could be factors influencing its widespread adoption. Future studies should focus on evaluating the cost-effectiveness of EDUG-guided PICC placement and explore ways to streamline its implementation to make it more accessible and sustainable in diverse healthcare settings.

Nonetheless, this study has certain limitations. It is a single-center retrospective study, which may be subject to selection bias and information bias. Some complications were recorded passively, which may have led to an underestimation of mild adverse events. Furthermore, the study did not include an economic analysis, and therefore the cost-effectiveness of EDUG-guided placement could not be assessed. In addition, while we performed statistical adjustments for potential confounding factors, such as age, gender, BMI, and tumor type, residual confounding factors, such as variations in chemoradiotherapy regimens, comorbidities, and other unmeasured variables, may still influence the outcomes. These factors were not fully controlled for, and their potential impact on complications needs to be considered. Future multicenter, prospective, randomized controlled trials are needed to validate the generalizability and economic feasibility of EDUG-guided tunneled PICCs across various cancer types and treatment settings, with more comprehensive control of confounding factors to minimize potential biases.

In conclusion, the EDUG-guided tunneled PICC demonstrates favorable safety and efficacy in cancer patients, particularly in reducing infection and complication rates and prolonging indwelling time. This technique holds promise as a preferred option for PICC placement and warrants broader adoption and refinement in clinical practice.

## 5. Conclusion

This study demonstrates that the EDUG-guided tunneled PICC offers significant advantages over the traditional PICC approach in cancer patients, including a higher first-attempt success rate, lower incidence of catheter-related infections and complications, and a significantly prolonged indwelling time. By optimizing the catheter pathway through subcutaneous tunneling, this technique enhances catheter fixation and reduces skin tension, contributing to improved biocompatibility and patient compliance. As an innovative method of catheterization, the EDUG approach significantly improves the safety and efficacy of PICC use and is particularly suitable for oncology patients requiring long-term intravenous therapy. Although further prospective studies are needed to validate its broader applicability and cost-effectiveness, the present findings provide strong evidence to support the clinical implementation of this technique in the cancer care setting.

## Author contributions

**Conceptualization:** Juan Zeng, Yi Zhang, Huili Xu.

**Data curation:** Juan Zeng, Yi Zhang, Huili Xu.

**Formal analysis:** Juan Zeng, Huili Xu.

**Investigation:** Juan Zeng, Yi Zhang, Huili Xu.

**Methodology:** Juan Zeng, Yi Zhang, Huili Xu.

**Supervision:** Juan Zeng, Yi Zhang, Huili Xu.

**Validation:** Juan Zeng, Huili Xu.

**Visualization:** Juan Zeng, Yi Zhang, Huili Xu.

**Writing** – **original draft:** Juan Zeng, Huili Xu.

**Writing** – **review & editing:** Juan Zeng, Huili Xu.
